# Genetic testing for Parkinson’s disease in clinical practice

**DOI:** 10.1007/s00702-023-02612-x

**Published:** 2023-03-16

**Authors:** Thomas Gasser

**Affiliations:** Tübingen, Germany

**Keywords:** Parkinson’s disease, Gene mutations, Disease mechanisms, Synuclein, Glucocerebrosidase, LRRK2

## Abstract

The identification of disease-causing mutations or strong risk factors for Parkinson’s disease in genes encoding proteins such as α-synuclein (SNCA), leucine-rich repeat kinase-2 (LRRK2), or glucocerebrosidase (GBA1) has led to a better understanding of the different components of disease pathogenesis. Many gene and mutation-specific targeted disease-modifying treatments are under development and several studies are under way. It is, therefore, important to raise awareness among patients and their families and to offer genetic testing, at least to those patients who are considering to participate in innovative trials.

## Introduction

Genetic research of the past two decades has greatly changed our understanding of neurodegenerative disorders, including Parkinson’s disease (PD). New genetic discoveries have provided insight into the molecular sequence of events leading to these complex disorders, including the aggregation of specific proteins prone to conformational changes, as well as lysosomal and mitochondrial dysfunction. There is evidence emerging that at least some of the pathways identified in the rare genetic forms of PD are also important in the common sporadic disorder.

Until recently, the consequences of this progress in genetic research have remained largely academic and restricted to improved genetic counseling in autosomal-dominant forms of the disease. Even this was of limited value, because penetrance of most PD-related mutations is incomplete, so predictive accuracy in individual cases is often low, and clear therapeutic consequences were lacking altogether.

In the past years, however, this is beginning to change. The understanding of the disease-associated molecular events and pathways, for example the role of seeding and aggregation of α-synuclein, of lysosomal protein degradation or mitochondrial maintenance, integrity. and clearing is beginning to allow the development of new disease-modifying treatment approaches that can be targeted specifically to patients with relevant molecular defects. First clinical trials using these treatments in genetically stratified patient populations are under way and more are being planned.

Although only a minority of patients will at present be eligible for these trials, genetic testing today is justified for all patients who would consider participation in one of those innovative treatment trials.

Different methods of genetic testing are currently available and the choice of the method depends on a number of considerations. If a mutation in one of the major PD genes described below can be suspected with a relatively high level of confidence, based on the clinical presentation and family history, direct Sanger sequencing of a particular gene may still be the most straightforward and economical strategy. In all other cases, either diagnostic panel sequencing of multiple relevant genes or clinical exome or genome sequencing may be performed, usually based on the specific expertise of the diagnostic laboratory.

## Autosomal dominant forms of Parkinson disease

### PD caused by mutations of the gene for α-synuclein (SNCA)

The discovery of the first PD-causing mutation in 1997 (Polymeropoulos et al. 1997) initiated a new era in PD research. The mutation was identified in a large family (referred to as the “Contursi kindred”) with an autosomal-dominantly inherited form of parkinsonism in a gene (the gene is abbreviated as *SNCA*, the protein as αSYN), with a single base-pair change from alanine (A) to threonine (T) at position 53 of αSYN (A53T) causing the disease with high, but not complete, penetrance. This discovery was soon followed by the observation that αSYN is the main constituent of the characteristic intracytoplasmic inclusions, long considered to be a hallmark of PD in general, the Lewy body (Spillantini et al. 1997). This finding has put αSYN at the center of the current understanding of the molecular pathogenesis of PD, although only very few further point mutations in the SNCA gene have been found so far, and thus are responsible only for a very small minority of cases.

Patients with mutations in SNCA, particularly the A53T mutation first identified in families of Greek origin, have a relatively early disease onset, often in their mid-forties, rapid progression of their motor symptoms, and early and severe cognitive impairment. The same phenotype of a “malignant” form of PD is seen in patients with triplications of the SNCA gene, leading to a doubling of the transcribed aSYN protein.

#### Molecular pathogenesis

Further insight into the molecular pathogenesis came from the discovery that multiplications of the wild-type *SNCA* gene (Singleton et al. 2003) can also lead to dominantly inherited PD with a dose-dependent effect. Patients with triplications have an earlier onset and more severe disease than those with duplications, who resemble those with sporadic PD. This result shows that even the wild-type protein can be pathogenic if present in excessive concentrations. Thus, the currently favored hypothesis states that either specific amino acid changes in αSYN with a subsequent alteration of its physicochemical properties or the overexpression of the wild-type gene may lead to an increased formation of β-pleated sheet conformation, which then leads to the formation of oligomeric and fibrillar aggregates, eventually resulting in neuronal dysfunction and cell death. This sequence of events has at least partially been recapitulated in animal models.

#### Consequences for counseling and treatment

PD caused by mutations in the SNCA gene is exceedingly rare, so genetic screening can be restricted to individuals with the characteristic picture of a relatively early disease onset, a positive family history compatible with autosomal-dominant inheritance and a “diffuse-malignant” clinical phenotype (Fereshtehnejad et al. 2015). However, it is important to build clinical “trial-ready cohorts” of mutations carriers, because due to the high penetrance of the mutation and the relatively severe phenotype, there is a major unmet need and this group may be particularly suited to explore the potential of anti-aggregation treatments even in pre-symptomatic individuals.

### PD caused by mutations in the LRRK2 gene

While *SNCA* point mutations or multiplications are rare, mutations in the gene for the leucine-rich repeat kinase-2 (*LRRK2)* were found to be a much more common cause of PD, accounting for 3–15% of familial cases, and for about 1–2% of seemingly “sporadic” cases in individuals of European descent (Zimprich et al. 2004). An even higher prevalence of a specific founder mutation, pG2019S, was reported to occur in up to 20–40% in Ashkenazi Jewish and North African Arab populations, in both sporadic and familial cases (Ozelius et al. 2006). The observation of this mutation in sporadic PD indicates reduced penetrance, which may vary, depending on the mutation and the population, between 30 and 80% (Marder et al. 2015; Ruiz-Martinez et al. 2010; Lee et al. 2017). This has to be taken into account in genetic counseling of patients and their families. Cases with *LRRK2* mutations generally have a similar age of onset as sporadic forms of PD (average about 59 years), but a more benign course with less cognitive impairment (Healy et al. 2008). Response to dopaminergic treatment is usually good, and typical fluctuations develop over time, so the general recommendations for symptomatic treatment apply to this subgroup.

Typical αSYN pathology with Lewy bodies and Lewy neurites is found in many but not all cases, and PD patients with LRRK2 mutations have also been reported to have pure nigral degeneration without Lewy bodies or even tau pathology.

#### Molecular pathogenesis

LRRK2 is a large multi-domain protein that contains several functionally important regions, including a “Ras of complex” protein (ROC) GTPase, which most likely functions as an intramolecular signaling mechanism, and a serine–threonine kinase domain. A group of intracellular signaling molecules called Rab proteins (“Ras-related in brain”) have recently been confirmed as substrates of LRRK2 kinase activity (Steger et al. 2017). It has been suggested that, by phosphorylating several Rab proteins, LRRK2 may be involved in presynaptic vesicle trafficking, cytoskeletal dynamics, autophagy, and/or the maintenance of the nuclear architecture. The way in which *LRRK2* mutations cause PD is also not clear, but there is evidence that increased kinase activity of the mutated LRRK2 that can also be measured in peripheral blood cells in humans (Fan et al. 2018), may contribute to the toxicity of pathogenic LRRK2 mutants.

#### Consequences for counseling and treatment

Based on this hypothesis, several CNS-penetrant, selective, ATP-competitive, small-molecule LRRK2 kinase inhibitors, have been developed. One of them, DNL201, has been shown to inhibit LRRK2 kinase activity and improved lysosomal function in preclinical cellular and animal models of the disease. In phase 1 and 1b clinical trials, DNL201 inhibited LRRK2 and was well tolerated in 122 healthy volunteers and in 28 patients with PD (Jennings et al. 2022), which led to the initiation of an international phase 2 clinical trial with the compound BIIB122, which is related to DNL201 (NCT05418673).

There is some evidence that LRRK2 kinase activity may also be of importance in sporadic PD, so DNL201 is also tested in patients without pathogenic LRRK2 mutation. Screening for LRRK2 mutations could, therefore, be recommended in all PD patients who are open for innovative treatment trials.

### PD with mutations in the GBA1 gene

Heterozygous mutations in the GBA1 gene, a gene causing the well-known autosomal-recessive lysosomal storage disorder Gaucher’s disease when mutated in a homozygous or compound-heterozygous state, have been identified as a strong risk factor for late-onset PD, increasing the risk for PD, depending on the severity of the mutation, by 3–15-fold (Sidransky et al. 2009). In other words, penetrance of a pathogenic GBA1 mutation is markedly reduced to 10–30%, with the clear majority of mutation carriers remaining unaffected even at advanced age, at least in the Ashkenazi Jewish population, where the relatively mild N370S mutation predominates (Alcalay et al. 2014). On the other hand, there is little doubt that GBA mutations are a major driver of the disease, as carriers clearly have a more severe disease course with relatively early and severe cognitive and autonomic disturbances, depending on the severity of the mutation (Brockmann et al. 2015).

More than 400 mutations, mostly missense mutations, have been described to be pathogenic in Gaucher’s disease. It is assumed that most, if not all, of these mutations will also increase the risk for PD in a heterozygous state (Do et al. 2019). On the other hand, there is at least one variant of the GBA gene, E326K, which does not cause Gaucher’s disease, but which is clearly associated with an increased PD risk (Sidransky et al. 2009). There seems to be a clear correlation of the parkinsonian phenotype and metabolic measures of GCase deficiency with the severity of the mutation (Lerche et al. 2021). Two of the most common mutations, L444P and N370S, are categorized as severe and mild, based on their association with different forms of Gaucher’s disease (the neuronopathic and non-neuronopathic forms, respectively).

#### Molecular pathogenesis

The GBA1 gene encodes the lysosomal enzyme glucocerebrosidase (GCase). Different mutations may be disease causing in different ways. It is thought that at least some mutations lead to inadequate intracellular transport of the encoded protein to its target organelle, the lysosome, leading to inadequate macroautophagy, which may in turn favor the accumulation of misfolded αSYN (Mazzulli et al. 2011). Other mutations may directly influence enzymatic activity of GCase.

An interesting aspect of GBA-associated PD is that the development of cognitive impairment and dementia appears to depend largely on the relatively pronounced spreading of αSYN pathology and less on concomitant amyloid-beta pathology, as is often the case in sporadic PD. In sporadic PD, but not in GBA-associated PD, an “amyloid-profile” (low CSF abeta and elevated pTau) is a major risk factor for the development of cognitive impairment (Lerche et al. 2017, 2019).

#### Consequences for counseling and treatment

Knowledge of a GBA mutation in a given PD patient, therefore, has consequences for planning treatment and care. Early and progressive cognitive impairment needs to be monitored and treated appropriately. As cognitive impairment increases the risk of psychiatric dopaminergic side effects, this has to be taken into account. Also there has been a report suggesting that DBS may lead to further cognitive impairment in patients with GBA-PD (Pal et al. 2022a). However, Weill and colleagues (Weill et al. 2022), while generally sharing the concern, pointed out that the un-operated control group in this study was taken from the large longitudinal observational PPMI study, which may introduce a bias for milder disease courses. So, no clear conclusion is possible at this time and further longitudinal well-designed studies on the cognitive outcome depending on treatment strategies in genetically characterized sub-cohorts with different GBA mutations are necessary (Pal et al. 2022b).

Due to the high prevalence of GBA mutations, roughly 10% of PD patients in Germany and 30% of the Ashkenazi Jewish population carry a GBA1-mutation, irrespective of family history—in otherwise typical late-onset PD and due to the relatively severe phenotype of GBA-associated PD, gene and mutation-specific treatments are under development.

A first large clinical trial was performed under the hypothesis that reducing one of the natural substrates of the GCase enzyme, glucosylceramide by inhibiting its synthesis with a CNS-penetrant glucosylceramide synthase inhibitor, venglustat (“substrate reduction”). In part 1 of the phase 2 MOVES-PD trial (NCT02906020), patients with heterozygous GBA1 mutations were treated with escalating doses and followed up to 36 weeks. Overall, the drug was well tolerated and over the treatment period, venglustat exposure in plasma and cerebrospinal fluid (CSF) increased, and GL-1 levels decreased in a dose-dependent manner, indicating that venglustat showed favorable safety and tolerability and target engagement was achieved in CSF (Peterschmitt et al. 2022). However, no effect on the progression of PD was seen in an interim analysis of part 2 of the trial, so the company decided to stop the trial. Further analyses of the data will show if an effect can be seen in certain subgroups of patients.

**Fig. 1:**
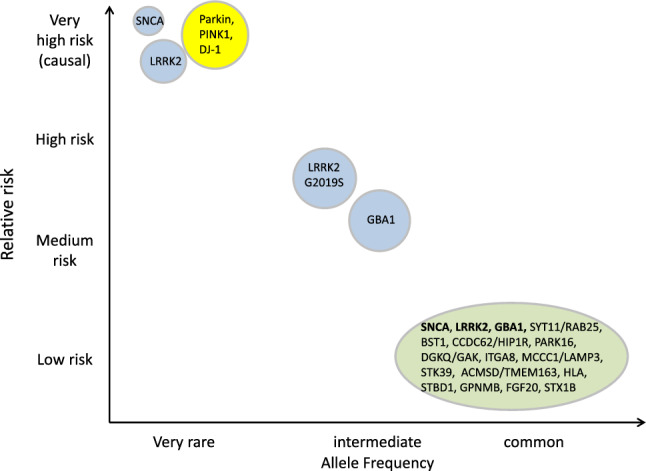
Genetic architecture of Parkinson’s disease (PD). Rare mutations in several genes confer a high or very high risk to develop the disease, they can be considered as “causative”, while a large number of common risk variants have a small, but measurable effect on disease risk. Some variants with intermediate frequency, for example the LRRK2 G2019S variant or variants in the GBA1 gene are strong “driver” risk variants of moderate effect strengths. Modified from Manolio et al. (2009)

Other approaches to specifically target the pathogenic mechanisms of GBA-associated PD to modify the disease course include the use of ambroxol, a compound that has been used as an over-the-counter mucolytic agent and that has been found to enhance GCase activity, probably by acting as a molecular chaperone improving intracellular delivery of the protein to the lysosome, and to reduce αSYN levels in several in vitro and in vivo models. A clinical phase 2 trial (NCT05287503) is currently under way (Fig. 1).

Another promising approach, particularly early in the course of the disease, would be GBA1 gene replacement therapy using an AAV viral vector (Abeliovich et al. 2021). A Phase 1/2a clinical trial (NCT04127578) is currently being conducted to evaluate safety, tolerability, immunogenicity, biomarkers, and clinical efficacy measures following intracisternal administration in escalating doses.

### Autosomal recessive forms of Parkinson disease

In 1998, the identification of a first gene mutated in an autosomal-recessive form of PD, autosomal-recessive juvenile Parkinson’s disease (ARJP), was reported (Kitada et al. 1998) in patients from Japan. The age at onset in ARJP cases is usually below 40 years, but the disease can sometimes start even in the second decade of life, with a motor deficit very similar to that of sporadic PD. Nevertheless, about 20% of patients with bi-allelic mutations have disease onset between 40 and 60 years of age, while onset later than age 60 is very rare. The first ARJP gene was named parkin (PRKN). The gene encodes an E_3_ ubiquitin ligase. Later, two other genes causing similar early-onset recessive forms of PD were identified: PINK1 and DJ-1 (Blauwendraat et al. 2020).

In addition to its early and sometimes “juvenile” onset, patients with ARJP often have dystonic symptoms, e.g., a dystonic gate disorder, at onset. Very brisk reflexes sometimes suggest another diagnosis, while the typical prodromal symptoms of α-synucleinopathies, such as hyposmia and REM-sleep behavior disorders, are much less common in the recessive forms of early-onset PD. In the course of the disease, a severe motor syndrome with fluctuations in response to levodopa treatment predominates, while cognitive impairment is rare and less severe, compared to sporadic PD. There is still some controversy concerning the neuropathology of ARJP. While typical Lewy pathology is not found in most patients with juvenile parkinsonism with parkin mutations (Schneider and Alcalay 2017), there have been individual reports of PRKN-mutated patients (often with unusually late onset) and a single autopsy of a case with PINK1-related ARJP that did show Lewy pathology (Samaranch et al. 2010).

#### Molecular pathogenesis of ARJP

Flies deficient in parkin and PINK1 were found to have mitochondrial dysfunction, with PINK1 apparently acting upstream of parkin in the same pathway. A currently favored mechanistic model holds that parkin is recruited from the cytoplasm to damaged mitochondria, where it is phosphorylated by the mitochondrial kinase PINK1, and subsequently ubiquitinates other mitochondrial membrane proteins, including for example VDAC1, which is the signal to initiate the elimination of dysfunctional mitochondria through a specific form of autophagy, called mitophagy (Geisler et al. 2010). As damaged mitochondria produce a high amount of toxic oxygen radicals, a failure of their clearance would lead to increasing cellular damage. These findings are particularly interesting in light of earlier findings identifying mitochondrial dysfunction as a possible etiological factor in sporadic PD.

#### Consequences for treatment and counseling

At present, there is no specific mutation-directed treatment of patients with ARJP carrying mutations in one of the identified genes. However, it is often comforting for patients with this disease to learn that in a recessive disease (presuming non-consanguinity), the risk for children to develop the same disorder is actually very low. While children are obligatory heterozygous carriers of pathogenic mutations in the respective genes, their risk to develop PD is probably, if at all, only very slightly above population background. Patients also often feel reassured to learn that cognitive symptoms are unusual in recessive PD and that progression is usually very slow. All medical and surgical treatments, including deep brain stimulation, are highly effective.

Based on the strong evidence that mitochondrial dysfunction plays an important role in the pathogenesis of recessive PD, a study has been initiated in genetically stratified subgroups of Parkinson's disease patients (PD) with homozygous or heterozygous mutations in one of the relevant genes or with an enrichment of risk variants in mitochondrial genes, who might benefit from treatment with the “mitochondrial enhancer” coenzyme Q10 (156 mg coenzyme Q10/d [QuinoMit Q10^®^ Fluid] over 6 months (Prasuhn et al. 2019). Magnetic resonance spectroscopy imaging will be used to quantify increased energy supply objectively. The study was registered at the German Clinical Trial Registry (DRKS, DRKS00015880).

### Relevance of genetic findings for sporadic Parkinson’s disease

The discovery of families in whom a disease, which is clinically and neuropathologically within the spectrum of Parkinson’s disease has led to enormous progress in our understanding of the distinct molecular pathogenesis of different forms of the disease, with the consequence that gene and mutation-specific treatments for several of these are being developed. In some, clinical trials have already been initiated.

Thus, the question arises, if this progress has any relevance for the majority of PD patients, i.e., depending on the population to the 70–90%, in whom no causative mutation or strong genetic driver variant can be identified.

In these cases, it is generally assumed that the disease is caused by an interplay of numerous, relatively common but low-effect genetic risk and protective variants with environmental influences on the background of an aging brain (Blauwendraat et al. 2020). In genome-wide association studies, more than 80 of these risk loci have been identified (Nalls et al. 2019). The frequency of individual risk variants can be as high as 30 or 40% in a population, but the effect size (i.e., the relative risk for a carrier of the risk, as opposed to the alternative “protective” variant) is in the range of 1,5 or less. The cumulative effect on disease risk in the quartile of individuals carrying the most risk variants is increased about fourfold over those with the lowest number (Nalls et al. 2019). However, at least some of those small effect, high prevalence risk variants appear to reside in genomic regions carrying also genes with strong risk variants or disease-causing mutation, in particular SNCA, LRRK2, and GBA. This observation has given rise to the hypothesis that genetic variants of different effect strengths, called “pleomorphic risk variants” in the same genes influence disease risk by distinct mechanisms (Singleton and Hardy 2011). Coding mutations in the genes themselves will usually alter gene function, as described above, while common risk and protective variants, which reside in regulatory regions of the gene, influence gene expression. It is, therefore, plausible that causative treatments developed for rare patients with monogenic forms of PD or harboring strong driver variants such as those in the GBA gene will eventually also benefit patients with sporadic PD, possibly stratified for specific combinations of common genetic risk variants.

Therefore, genetic testing has come of age and should at least be offered to interested patients.

## Data Availability

The dataset(s) supporting the conclusions of this article is(are) available in the MDSgene mutation repository [https://www.movementdisorders.org/MDS/Resources/MDSGene.htm].
